# Potential diagnostic and drug target markers in glioblastoma

**DOI:** 10.1038/s41598-024-57752-1

**Published:** 2024-03-27

**Authors:** Hina Ahsan, Muhammad Asghar, Shaukat Iqbal Malik

**Affiliations:** 1 Department of Bioinformatics and Biosciences, Faculty of Health and Life Sciences, Capital University of Science and Technology (CUST), Islamabad, 44000 Pakistan; 2 Department of Biology, Lund University, Lund, Sweden; 3 Department of Healthcare Biotechnology, Atta-Ur-Rahman School of Applied Biosciences (ASAB), National University of Sciences and Technology (NUST), Islamabad, H-12 Pakistan

**Keywords:** Glioblastoma, Transcriptomic profiling, Differential gene expression, Anti-cancer peptides, Biomarkers, Cancer, Drug discovery, Biomarkers, Oncology

## Abstract

Glioblastoma multiforme (GBM) IDH-wildtype is the most prevalent brain malignancy in adults. However, molecular mechanisms, which leads to GBM have not been completely elucidated. Granulocyte colony-stimulating factor (GCSF), Granulocyte colony-stimulating factor receptor GCSFR, and Signal transducers and activators of transcription 3 (STAT3) have been involved in the occurrence and development of various cancers, but their role in GBM is little known. Herein, we have investigated the gene and protein expression of GCSF, GCSFR, and STAT3 in 21 tissue biopsy samples and also in tumor associated normal tissue (TANT) samples derived from glioblastoma patients, which revealed significantly differential expression of these genes. To validate our findings, we performed a comprehensive integrated analysis of transcriptomic and proteomic profiling of respective genes by retrieving GBM RNA-sequence data from Genome Atlas Databases. GO and KEGG analysis revealed enrichment in disease-related pathways, such as JAK/STAT pathway activation, which were associated with GBM progression. We further performed computational docking analysis of potential drug candidate Nisin against GCSF, and the results were validated in vitro through cytotoxic activity assay using a human glioblastoma cell line SF-767 in a dose-dependent manner. Our comprehensive analysis reveals that GCSF augments glioma progression, and its blockade with anticancer bacteriocin peptide Nisin can potentially inhibit the growth and metastasis of GBM.

## Introduction

Glioblastoma multiforme (GBM) is the most lethal malignant brain tumour, with a 5-year survival rate < 3% and a median survival of fewer than 15 months^1^. Glioblastoma was recently classified as CNS WHO grade-4 IDH wildtype diffuse glioma with microvascular-proliferation and/or intertumoral-necrosis^2^. Although immunotherapy has transformed cancer treatment in recent years, GBM is an immunologically cold tumour typically resistant to this therapy due to the development of immune suppression mechanisms. Various studies have explored the intrinsic variables involved in tumour heterogeneity and progression^3^. However, tumour microenvironment dynamics are poorly understood and demand extensive study exploring the role of cytokines in the tumour microenvironment, either promoting tumour growth or suppressing its malignant aspects^4,5^. Signal transducer and activator of transcription 3 (STAT 3) pathway is one of five critical pathways disrupted in human glioblastoma**.** In this context, we hypothesized that granulocyte-colony stimulating factor (GCSF), a potent mitogen for various cell types, may have a role in GBM by regulating the phosphorylation state of STAT3^6^. While the exact mechanisms linking GCSF and STAT3 phosphorylation in GBM warrant further investigation, several plausible pathways can be considered. The ability of GCSF to phosphorylate STAT3 is consistent with its capacity to activate Janus kinase (JAK) family members. STAT3 is known to be activated by the JAK kinases when it is phosphorylated at tyrosine residues. The subsequent phosphorylation and dimerization of STAT3 following GCSF-induced JAK activation may promote STAT3 nuclear translocation and transcriptional activity^7^. The pathogenesis of GBM may then influence the expression of genes involved in cell survival, proliferation, and immune evasion^8^. In multiple cellular preferences, cytokines—including GCSF—have been found to activate STAT3. This activation frequently occurs because of JAK kinase recruitment and activation, which then phosphorylate and activate STAT3. It has been demonstrated that cytokines and STAT3 signaling pathways interact in a variety of cell types, including GBM. Additionally, GBM has a complex tumor microenvironment that contains immune cells. GCSF was first recognized as a leukemic differentiation factor and granulocyte stimulator, regulating granulocyte, macrophage, and hematopoietic progenitor cell proliferation, maturation, and survival etc.^9^. The recruitment of immune cells by GCSF may indirectly affect STAT3 phosphorylation. Immune cells like granulocytes are mobilized and undergo differentiation as a result of GCSF. These immune cells can release cytokines and create inflammatory microenvironment in GBM. Through paracrine actions, such inflammatory settings can increase STAT3 signaling in GBM cells^10^. According to the growing evidence, GCSF (CSF3) and its receptor GCSFR (CSF3R) are overexpressed in numerous malignancies, including melanoma, non-small cell lung cancer, bladder, prostate, and brain tumours^11,12^.

Herein, we investigated the expression of GCSF, GCSFR, and STAT3 genes in 21 tissue biopsy samples and also in tumor associated normal tissue (TANT) samples derived from glioblastoma patients, which revealed differential expression of these genes. The biopsy specimens of malignant glioblastoma were found with an elevated level of GCSF and its receptor resulting in tumour progression. We further performed a comprehensive integrated analysis of transcriptomic and proteomic profiling of GCSF, GCSFR, and STAT3 by retrieving GBM RNA-sequence data from genome atlas databases. Moreover, increased GCSF (CSF3) expression associated with tumour grade, mutational status, immune infiltration patterns and the concomitant presence of anomalies in the JAK/STAT signaling pathway responsible for the favorable tumor microenvironment were reported. Various computational tools are used that supplemented data resource integration using the Genomic R/Bioconductor package incorporating web servers to evaluate data sets. The findings suggested that increased GCSF expression contributes to glioblastoma onset and progression**.** Therefore, the hypothesis that GCSF regulates STAT3 phosphorylation in GBM is supported by overexpression of GCSF, STAT3's significance in GBM biology, and documented cytokine-STAT3 interactions. As a result, we predicted the Nisin bacteriocin peptide as the possible drug candidate that may dampen the glioblastoma aggressiveness by inhibiting overexpressed GCSF. According to previous studies, nisin exhibits anticancer abilities via inhibiting cell growth and inducing apoptosis, with additional mechanisms. Nisin has been suggested to inhibit GCSF-induced signaling pathways in cancer cells, making it a promising candidate for targeted therapy in glioblastoma^13^. In sum, the analyses provided new insights into molecular mechanisms underlying GBM etiology, identifying GCSF driving tumorgenicity, potential biological and clinical significant markers for therapeutic prognostic and diagnostic implications.

## Materials and methods

**Figure 1:**
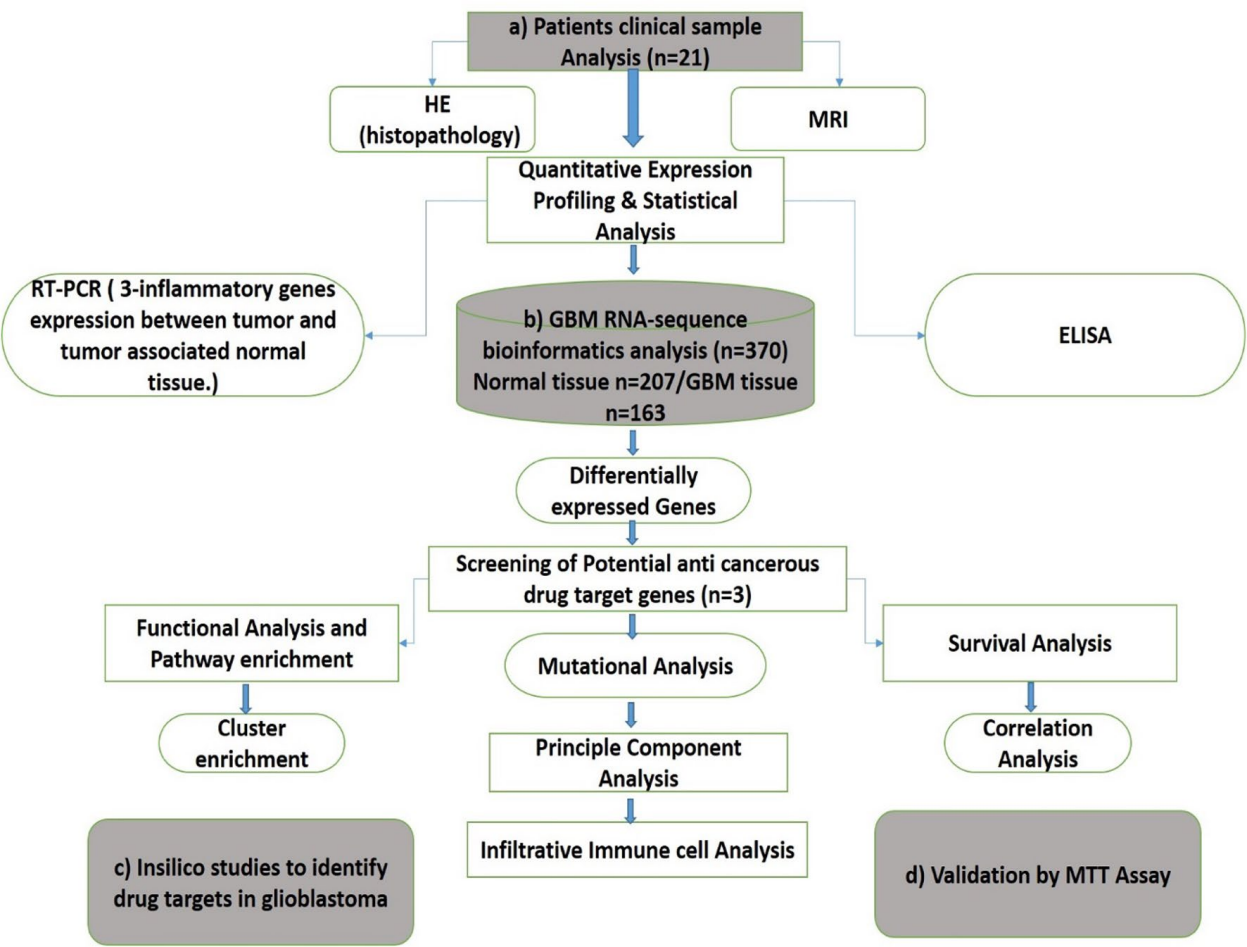
Schematic illustration to identify key drug target associated with glioblastoma.

An overview of workflow has been summarized and consists of the following four sections; (a) Patients clinical samples, (b) GBM RNA-sequence bioinformatic analysis, (c) Insilico studies to identify drug targets in glioblastoma and (d) validation by MTT Assay as shown in Fig. 1.

### Clinical samples analysis

#### Ethical approval and resource sharing

The Capital University of Science and Technology (CUST), Islamabad, Pakistan, research and ethical committee gave its approval for the study, which was carried out in accordance with the Declaration of Helsinki (Ref: BI&BS/ERC/19–2 and September 23, 2019). "All of the patients agreed verbally and in writing to have their data used for research. From several surgery departments of public sector tertiary care hospitals in Pakistan, biopsy samples of 21 glioblastoma patients (13 men, 8 women, median age 50 ± 13 years) who underwent brain surgery between January 2018 and December 2021 were collected. Prior to sample collection, none of the study individuals had undergone radiation therapy or chemotherapy. In the current study the sample size based upon the burden of high-grade glioma assessed by counting the total number of glioblastoma (CNS WHO grade 4) IDH wildtype diagnosed cases leading to surgical procedure within a given time frame and geographic region. Factors such as patient availability, ethical considerations, and specific biomarkers or endpoints affecting the power analysis were also considered. However the effect size, desired power and significance level, heterogeneity, availability and feasibility, and power analysis were applied by using G*Power, R with the power package, and SAS^14^.

#### Inclusion and exclusion criteria

In the current study selection criteria, patient demographics and characteristics such as age, gender, tumor location, and molecular subtype, has been considered. The study primarily focuses on Asian ethnicity. It includes participants from diverse ethnic backgrounds in Pakistan, including Punjabi and Pashtun. The study includes both men and women to explore gender-specific differences in glioblastoma incidence and outcomes. However Inclusion criteria were considered by newly diagnosed glioma tissue biopsies based on Immunohisto- chemical confirmation and radiological findings (CNS WHO grade 4, IDH wildtype) that were amenable to surgical resection, a Karnofsky Performance Score (KPS) 60%, Histopathological verified diagnosis after the CNS5 WHO classification, adequate liver function, and adequate renal function. Eligible ages for study including the patients with: 10 years and older (Adult, older Adult), all gender eligible were for current study, control biopsies = Tumor Associated Normal Tissue (TANT) minimum ≈ 11, Non-Probability sampling method was applied. Both type of glioma patients whether underwent chemotherapy or not was applied^15^. Exclusion criteria included acute infections requiring active treatment, unstable or serious coexisting illnesses (such as pulmonary, cardiac, or other systemic diseases), known immunosuppressive diseases, positive HIV or hepatitis B serology, a history of an autoimmune disease, or prior history of other malignancies. Any kind of brain metastasis from a body malignancy, Pregnancy, second primary malignancy (cervical cancer that is still in situ, prior cancer that was treated more than five years before to enrollment without recurrence), or T1 vocal cord cancer in remission Brain tumours other than gliomas such as gliomatosis cerebri are present. The size of the patient's glioma is less than 2 cm3. Ages under ten years, the influence of complex co-morbidity causing systemic illness in the study, and patients with incomplete or missing data regarding their demographics, tumour type, or tumor site^16^.

#### Tissue samples

The samples were obtained from patients with glioblastoma multiforme (GBM) through surgical resections, and tumor tissue samples were collected from the affected brain regions. The samples were sectioned into small fragments, (approximately 1–2 mm^3^ in size) preserved using formalin fixation and paraffin embedding and stored at ultra-low temperatures (− 80 °C) to maintain their integrity and prevent degradation. Ethical safeguards were also implemented to ensure responsible research practices^17^.

#### MRI imaging

Each patient was subjected to an intraoperative MRI scan for trajectory planning on a 3 Tesla MRI scanner (Siemens AG Healthcare, Erlangen) with the following criteria: FoV = 260 mm × 260 mm, voxel size = 1.03 mm^3^, and image matrix = 256 × 256.

#### Histopathology

Twenty-one Biopsy samples, approximately 1 mm^3^ in size were collected and using hematoxylin and eosin staining (HE stain), the purity of the tumour samples was assessed by a histopathologist to validate that each sample contained > 80% malignant cells.

#### Quantitative RT-qPCR analysis

In order to reduce degradation by ubiquitous DNases and RNases, bio specimens of glioblastoma designated for genomic analysis were micro dissected and kept in the nucleic acid stabilising reagent RNA later (Sigma-Aldrich). Specimens were immediately frozen in liquid nitrogen after ablation and stored at -80 °C until RNA extraction. Total RNA was extracted using the TriZol reagent (Thermo Fisher Scientific). Superscript II reverse transcriptase (Invitrogen, Paisley, UK) was used to create cDNA by cDNA synthesis kit (Thermo Scientific, Cat, No: K1622) and the SYBR® Green Master Mix kit (Maxima SYBR Green/ROX qPCR Master Mix (2 ×) (Thermo fisher Cat No: K0221), was utilized for qPCR to amplify the specific PCR products of the three genes presented in this work (Thermoscientific, CA, USA). Using a Nano Drop One spectrophotometer from Thermo Fisher Scientific, the purity of each RNA sample was determined. Using the 2 –ΔΔCt technique and β -actin as the reference gene, the mRNA expression of each gene was evaluated. The primer used for GCSF (Forward Primer GTGCCACCTACAAGCTGTGC and Reverse Primer AAAGGCCGCTATGGAGTTGG) and GCSFR (Forward Primer AAGAGCCCCCTTACCCACTACACCATCTT and Reverse Primer TGCTGTGAGCTGGGTCTGGGACACTT), STAT3 (Forward Primer CATATGCGGCCAGCAAAGAA and Reverse Primer ATACCTGCTCTGAAGAAACT) and for β –actin (Forward Primer CATGTACGTTGCTATCCAGGC and Reverse Primer CTCCTTAATGTCACGCACGAT)^18,19^.

#### ELISA

Prior to protein extraction, Glioblastoma biopsy samples were sealed in sterile containers, snap-frozen until protein extraction and kept at -80 °C. Supernatants were slowly defrosted on ice. 96-well enzyme-linked Immunosorbent assays (ELISA) were used to measure 100 µl of supernatant. The concentrations of GCSF, GCSFR, and phosphorylated STAT3 were measured using protein-specific ELISA kits (Abcam ELISA kit USA) according to the manufacturer's procedure. Using the appropriate ELISA MAXTM Deluxe Set-in accordance with the manufacturer's guidelines, cytokine levels were assessed by Human GCSF ELISA Kit, Cat. No. (ab100524), Human GCSFR ELISA Kit Cat. No (ab267649) and Human STAT3 ELISA Kit Cat. No (ab264629). A spectrophotometer immediately measured the specific binding optical density at 450 nm^20^**.**

#### Cluster analysis of gene expression using hierarchical heat maps, principal component analysis (PCA), and uniform manifold approximation and projection (UMAP)

Qlucore Omics Explorer 3.8.9 was used to perform PCA, Hierarchical Heat Map Cluster and UMAP analysis with a cut of q-value (adjusted p-value) of < 0.05 for the expression of GCSF, GCSFR and STAT3 genes. The Euclidean distance was taken as the default distance technique, and complete linkage as agglomeration mode. After removing variables with high overall variance to reduce the impact of noise, the remaining variables were scaled and centered to have zero mean and unit variance before being visualized using PCA. The projection score was used to determine the ideal filtering threshold in order to keep N variables. We used Uniform Manifold Approximation and Projection (UMAP) on the normalized count data of three genes, and using the default settings (Benjamini-Hochberg) in Qlucore Omics Explorer 3.8.2^21,21,21^.

### Bioinformatic analysis of genome atlas databases of glioblastoma

#### Transcriptomic analysis

To investigate the differentially expressed genes (DEGs) in GBM patients, The Cancer Genome Atlas (TCGA), Genome Tissues Expression database (GTEx) and Gene Expression Profiling and Interactive Analyses GEPIA2 were used^23^. We retrieved expression data of 163 glioblastoma cases from TCGA and 207 normal brain tissues from GTEx. We validated differential analysis of GCSF, GCSFR, and STAT3 expression via GEPIA2 and depicted the results using a boxplot with log2 of transcript count per million representing the expression level of DEGs. Log2FC| Cutoff was applied to compute p-values. The log2FC| Cutoff value was adjusted at 1 with p-value cutoff at 0.01.

#### Survival analysis

We grouped glioblastoma multiforme (GBM) samples into high and low GBM classes based on the optimal cut-off using the R package surv Misc and the gepia-2 programme. A survival package was used to conduct a Kaplan–Meier analysis employing a log-rank test to investigate the relationship between GBM-associated genes expression under investigation and survival. A p-value of 0.05 or less was taken as statistically significant^24^.

#### Genomic landscape mutation analysis

Using a human proteo-genomics database, Active Driver DB, and the cBioportal database, we analyzed DEGs mutations^25^. These are utilized to detect protein post-translational modification (PTM) sites and to visualize and analyze multimodal cancer genetics. Gene alterations, a set of gene types, the association between gene mutations and the prognosis of GBM patients were assessed using cBioPortal based on the TCGA database. The significance level was set at p < 0.05^26^.

#### Infiltrative immune cell analysis

To investigate the infiltration of various immune cells and their clinical impact, the immune cell correlation analysis of GCSF was practiced using the immunedeconv package in R and CIBERSORTx method through TIMER2.0 server, integrating samples data from the TCGA and CGGA datasets. After setting batch correction, executing "Bulk mode," and selecting the quantile normalization algorithm, sample results were adjusted for purity where necessary, and correlations with Spearman's p < 0.05 were shown. Using the Wilcoxon rank-sum test, the differences between the two subgroups were determined^27^.

#### Gene enrichment ontology and protein interactions

Gene ontology studies were conducted utilizing the g: Profiler, David, and Funrich servers^28^. Significant signaling pathways and cellular components of differentially expressed genes were identified using GO. Protein–protein interactions (PPIs) were performed to explore differences in biological function. Metascape and STRING.v.10 were used to find the intrinsic interactions of the source gene^29,29,29^. Additionally, the functions of the target genes in GBM were retrieved from several databases, including PubMed, CTD, and OMIM^31^. Cytoscape software version 3.6 was used to illustrate the network to investigate the significance of source (DEGs) and target proteins in patients with glioblastoma^32,33^.

#### Pathway enrichment and integrated modelling

We investigated the pathway enrichment of DEGs using the Shiny GO tool and FunRich tool version 3.1.3 with p-values < 0.05 that were statistically significant^34^. The curation and mapping of potential biomarkers were performed using the Reactome, Kyoto Encyclopedia of Genes and Genomes (KEGG), and Wiki pathways^35^. PathVisio3 tool was utilized to reassemble the biological and signaling pathways of prospective biomarkers^36^.

### Molecular docking using nisin bacteriocin peptide complex

#### Retrieval of experimentally reported nisin and visualization

The crystal structure of the target protein, named GCSF (PDB: 5GW9), was obtained from the Protein Data Bank (www.rcsb.com). The sequence was retrieved from the Uniprot database^37^. Using Chimera software, water molecules and heteroatoms were removed from the PDB data. The potential anticancer Nisin bacteriocin peptide (PDB: 1WCO) was also retrieved from the Protein Data Bank (www.rcsb.com)^38^. The protein and drug library were generated using the Molecular Operating Environment (MOE) software. It was also used for scoring functions to predict the binding affinity of the Nisin bacteriocin peptide to its target, and to provide insights into potential antibacterial interactions. MOE was used for molecular modeling, employing algorithms to explore possible orientations and conformations of the ligand within the protein's binding site. To prepare proteins and ligands for docking, the protonate 3D technique in MOE was employed to add hydrogens, followed by energy minimization, electrostatic interactions, and solvation effects^39^. After minimizing energy, the AMBER99 force field was employed to eliminate additional unbounded structures. Seven optimal configurations were selected using the force field refinement method. Protein–protein docking studies of putative bacteriocins and their interacting proteins were validated by ClusPro^40^. UCSF chimera was used to analyze and visualize the docking results (www.cgl.ucsf.edu/chimera/download.html)^41^.

#### Model evaluation and validation

The Procheck tool confirmed the stereochemical correctness and overall structural geometry of the protein structure^42^. The Ramachandran plot statistics were used to assess the model's stability and validate the residues. In addition, the Ramachandran plot and Z-score analysis were performed on four high-resolution GCSF structures selected from the PDB database with PDB ID 5GW9^43^. Normal mode analysis (NMA) was also carried out to comprehend the stability and flexibility of the docked model. The iMod technique was used to calculate the degree of stability. The elastic network model, deformability, eigenvalue, and covariance matrix were computed^44^.

### Cytotoxicity assay

The cell viability percentage was evaluated using MTT assay. The Cells (human glioblastoma SF-767) were cultured in appropriate growth media and conditions until they reached the desired confluency or growth phase. In a 96-well culture plate, approximately 1 × 10^3^ cells of SF-767 glioblastoma cell line was seeded and allowed to adhere. The cells were treated with different dosage concentrations of Nisin (1, 5, 10, 30, 60 and 100 µg/mL) for 48 h at 37 °C. The cellular fraction was labelled with MTT solution (5 mg/mL in PBS) for 4 h after discarding the supernatant, followed by solubilization in 50 µL of dimethyl sulfoxide (DMSO). The plate also contained cells treated with PBS as a negative control. The absorbance of the solubilized formazan is measured using a microplate reader at a specific wavelength 570 nm (with 620 nm as a reference) to quantify the amount of formazan produced. The absorbance is proportional to the number of viable cells^45^.

### Statistical analysis

The clinical, radiological, and pathological data has been presented in a structured manner. Categorical variables were expressed as numbers and percentages, while continuous variables were represented as means ± standard deviations (SDs) or medians with interquartile range, depending on data distribution. Statistical comparisons were made using appropriate methods, such as the chi-squared test for categorical variables and Student's t-test for normally distributed continuous variables^46^. For RT-PCR and ELISA data, the mean ± SD from at least three independent experiments for statistical analysis were calculated by GraphPad Prism 9 software. The statistical significance using the chi-square test and two-tailed Student's t-test, and data normality was evaluated using the D’Agostino & Pearson test. Inclusion and exclusion criteria were applied While considering the patients cohorts with complete data as compared to excluding patients with missing or incomplete data based on predefined exclusion criteria. Statistical significance was determined at P < 0.05. Median overall survival (mOS) with 95% confidence intervals (95% CI) was calculated for different glioma subtypes and distinct molecular features. Kaplan–Meier curves illustrated overall survival, with log-rank tests indicating significant differences between groups. Data analysis was conducted using SPSS (version 26.0, IBM, USA), and graphs were generated using RStudio (PBC & Certified B Corp.®, USA)^47^. Batch effects were identified and mitigated through preprocessing and statistical methods. Following batch effect removal, intended analyses, such as differential gene expression and survival analysis, were performed to provide accurate and interpretable results^48^.

### Ethics statement

The Capital University of Science and Technology (CUST), Islamabad, Pakistan, research and ethical committee gave its approval for the study, which was carried out in accordance with the Declaration of Helsinki (Ref: BI&BS/ERC/19–2 and September 23, 2019). Informed verbal and written consents were obtained from all patients to have their data used for research. From several surgery departments of public sector tertiary care hospitals in Pakistan, biopsy samples of 21 glioblastoma patients (13 men, 8 women, median age 50 ± 13 years) who underwent brain surgery between January 2018 and December 2021 were collected. Prior to sample collection, none of the study individuals had undergone radiation therapy or chemotherapy.

## Results

### Clinical sample analysis

#### Gene expression signatures in distinct spatial regions of glioblastoma

**Figure 2:**
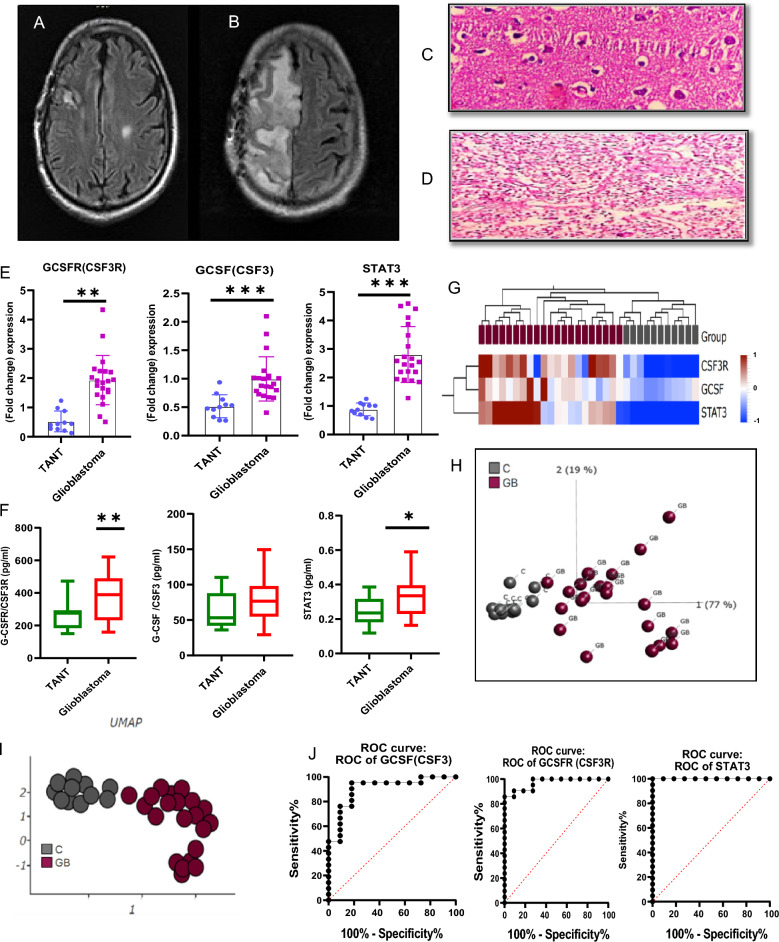
Quantification expression of glioblastoma patients. (**A**) Representative axial slices of T1-weighted MRI after contrast administration with small areas of patchy enhancement. (**B**) T2-weighted FLAIR obtained prior to stereotactic brain biopsy show a predominantly enhancing lesion within the left lobe with associated edema. (**C**) Histopathology image of TANT depicting the agglomeration of tumor cells around the periphery of necrotic regions, together with enhanced mitotic activity and vascular growth. (**D**) Hematoxylin and eosin staining (HE staining) also shows the arrangement of small hyperchromatic tumor cells in a parallel fashion, which resembles the arrangement in neuronal tumors. The images were retrieved using 40X magnification power. (**E**) Expression levels of the 3 candidate reference genes (GCSF, GCSFR, STAT3) in biopsy tissue of glioblastoma through RT-PCR. The statistical significance of differential expression is determined using distributions of gene expression levels by t-test at ** *p* < 0.01, and *** *p* < 0.001. As *p* values of less than 0.01 and less than 0.001 are denoted by two and three asterisks, respectively. The graphs were plotted with the GraphPad Prism 9 (Prism 9.5.0) software. (**F**) Enzyme Linked Immunosorbent Assay validated the expression of DEGs in glioblastoma patients by using the t-test. * *p* < 0.05 and** *p* < 0.01 respectively. (**G**) Heat Map and hierarchical grouping of glioblastoma samples based on three differentially expressed genes with the highest coefficient of variation across all samples. Rows are centered; unit variance scaling is applied to rows. Both rows and columns are clustered using correlation distance and average linkage. Heatmaps colored from blue to red brown according to Z-score scale − 1 to 1. (**H**) Unit variance scaling is applied to calculate principal components. X and Y axis show principal component 1 and principal component 2 that explain 77% and 19% of the total variance. (**I**) Uniform manifold approximation and projection (UMAP) analysis showed separate clusters in between C and GB. However, suggesting a high degree of heterogeneity for the tumor brain in individual patients of glioblastoma. (**J**) ROC curves show specificity of GCSF and GCSFR in the prognosis of glioblastoma**.**

GBM tumors were characterized by Magnetic resonance imaging of axial slide of T1-weighted MRI after contrast administration with small areas of patchy enhancement and T2-weighted FLAIR obtained prior to stereotactic brain biopsy show a predominantly enhancing lesion within the left lobe with associated edema in (Fig. 2A,B). Histopathology findings indicated that the HE staining of glioblastoma tissues had evident atypia and deeper staining as compared to adjacent tumor associated normal tissues which showed agglomeration of less tumor cells around the periphery of necrotic regions, together with some mitotic activity and vascular growth (Fig. 2C). While (Fig. 2D) showed a high proliferation index, localized necrosis, hyperchromatic tumor cells in a parallel fashion, and dense cellularity at 40X magnification power. Nonetheless, there may be instances in which combinations of many lineages contribute to the tumor burden. To evaluate the potential role of GCSF in glioblastoma, we quantified the expression of GCSF and its receptor (GCSFR) and STAT3. GCSF mRNA and protein expression was significantly increased in glioblastoma biopsy samples. We examined the gene expression of targeted genes among glioblastoma specimen sections within tumors and tumor-associated normal tissue (TANT). All tissue samples were initially cut from four regions of the specimen, but samples with sufficient RNA quality and quantity was subjected to RT-PCR analysis of gene expression (Fig. 2E). GCSF, GCSFR, and STAT3 exhibited increased expression in tumor tissue biopsy samples. GCSF and STAT3 also showed positive Spearman correlation in GBM biopsy tissues R = 0.20 and in TANT was R = 0.009 while the p-value was considered non-significant each. These quantitative expression data are also consistent with ELISA findings (Fig. 2F). These findings were validated by Intra- and Inter-Assay Variability factor by Evaluating variation within (intra-assay) and between (inter-assay) different runs or experiments^49^. As low Coefficient of Variation (CV) values indicate that repeated measurements of the same sample yield consistent results of RT-qPCR and ELISA and these method show high reproducibility based on low CV etc. lastly Linearity and Accuracy was also analyzed to validate the results by Constructing standard curves using known standards or controls with a high correlation coefficient (R-squared) these results also conducted measurements with an appropriate number of replicates to ensure statistical significance and robustness of these findings through t-tests, ANOVA, or regression analysis, by considering significant p-values^50^. Heat map and expression profile similarity demonstrated that all tumour samples clustered similarly, showing that the expression profile within a tumor specimen is preserved across the specimen with elevated gene expression. However there are alot of heterogeneity across the samples. This shows that the significant variation in gene expression occurs across samples of various grades of the tumor, followed by samples of the same grade, and finally, within a specific sample. Average linkage and correlation distance in 3 rows and 32 columns were grouped. Heatmaps showed that tumor cells have high expression of genes as compared to tumor associated normal tissue in respect to color range from blue to red brown according to Z-score scale − 1 to 1 (Fig. 2G). Principal component analysis (PCA) showed overall separation between the TANT and GBM patient samples. Rows are subjected to unit variance scaling, and SVD with imputation is employed to determine the major components. Principal components 1 and 2, which account for 77% and 19% of the total variance, are displayed on the X and Y axes, respectively (Fig. 2H) and uniform manifold approximation and projection (UMAP) also showed separation between control and diseased samples, specifically the TANT (marked as C) and GBM (marked as GB) were clustered separately. More over the GB showed a dispersed pattern of distribution with others patient group neighboring cluster and the C group showed very low variation, suggesting a high degree of heterogeneity among patients (Fig. 2I). The expression of GCSF and its receptor was confirmed by the receiver operating characteristic (ROC) curves for each of our predictive schemes in the categorization of patients based on their CNS WHO grade 4 malignancy and was also employed to classify glioblastoma based on the combination of three distinct feature types: histopathological, expression, and magnetic resonance imaging characteristics Similarly, the GCSF and GCSFR cut-off point was determined for which accuracy measures were derived from cross-tabulations (Fig. 2J).

### Global RNA-seq bioinformatic analysis

#### Variegated expression and prognostic Value of GCSF, GCSFR, and STAT3

**Figure 3:**
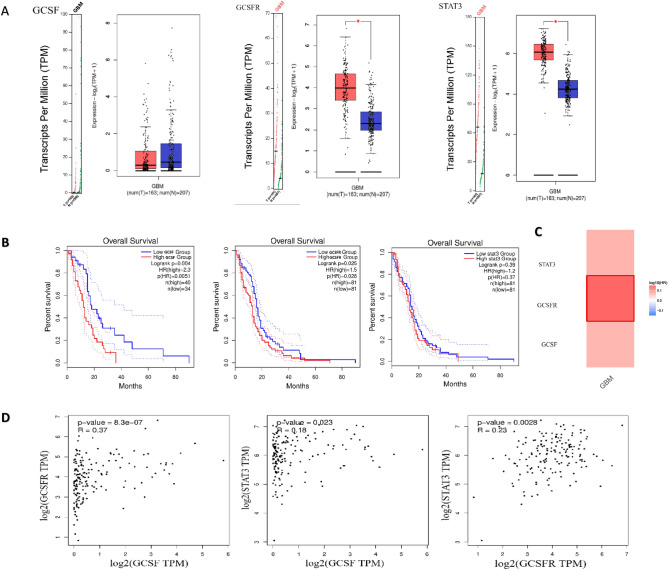
GCSF, GCSFR and STAT3 expression, survival analysis, heat-map and their correlation in glioblastoma. (**A**) Tissue gene expression according to the GEPIA2, TCGA and GTEX databases. GCSFR, GCSF, and *STAT3* show significant (*p < 0.01) differential expression between GBM and normal tissue. The cut-off value for log2FC (fold change) was 1. We used a p-value threshold of 0.01 and a jitter size of 4. TPM is an acronym for transcripts per million. (**B**) *Using the GEPIA2, TCGA, and GTEX platforms, Kaplan–Meier survival graphs were generated. The overall survival curve of several malignant tissues was investigated between a high expression group (red line) and a low expression group (blue line) of GCSFR, GCSF, and STAT3, using p* = *0.01 as the threshold for statistical significance.* (**C**) The survival heat map of hazard ratio (HR) indicates the prognostic impacts of highly significant expressed genes, e.g. GCSF log10 (HR) = 2.3, GCSFR log10 (HR) = 1.5 and STAT3 log10 (HR) = 1.2. (**D**) The correlation analysis of GCSF, GCSFR, and STAT3 has been depicted. GCSF and GCSFR revealed a high correlation with an R-value of 0.37.

To confirm our findings, we performed a comprehensive integrated analysis of transcriptomic and proteomic profiling of respective genes by retrieving GBM RNA-sequence data from genome atlas databases. Using the GEPIA2, TCGA, and GTEX integrated platform with the integration of R, we discovered that the expression of GCSFR and STAT3 was higher in GBM samples (n = 163) compared to normal brain tissues (n = 207) (Fig. 3A). However, GCSF expression was not significant, similar to our finding where GCSF expression was higher in GBM samples, but difference did not reached to log2FC (fold change). To study the link between the differential expression of GCSF, GCSFR, and STAT3 genes and GBM patient prognosis, we assessed the correlation between differential expression and overall survival with GEPIA2. It demonstrated that patients with a high expression of GCSF, GCSFR and STAT3 had a lower overall survival (Fig. 3B). The survival heat map of hazard ratio Log10 (HR) indicated the prognostic impacts of GCSF, GCSFR and STAT3 and compared the survival contribution of GCSF, GCSFR and STAT3 by using Mantel-Cox test. The hazard ratio values for GCSF, GCSFR, and STAT3 were 2.3, 1.5 and 1.2, respectively (Fig. 3C). The correlation between GCSF (CSF3) and GCSFR (CSF3R) was analyzed, and highly correlate in glioblastoma patients with an optimum cut-off median at significance level p = 0.05. The high correlation between GCSF (CSF3) and GCSFR (CSF3R) was considered significant and positive, with R-value of 0.37 (Fig. 3D), which demonstrated a positive correlation between OS and variation of expression. These results suggest that differential expression of GCSF, GCSFR and STAT3 impart a critical role in the prognosis of patients with GBM and may prove an appropriate survival predictor in these patients.

#### Genomic landscape alteration and frequency changes of GCSF in GBM patients

**Figure 4:**
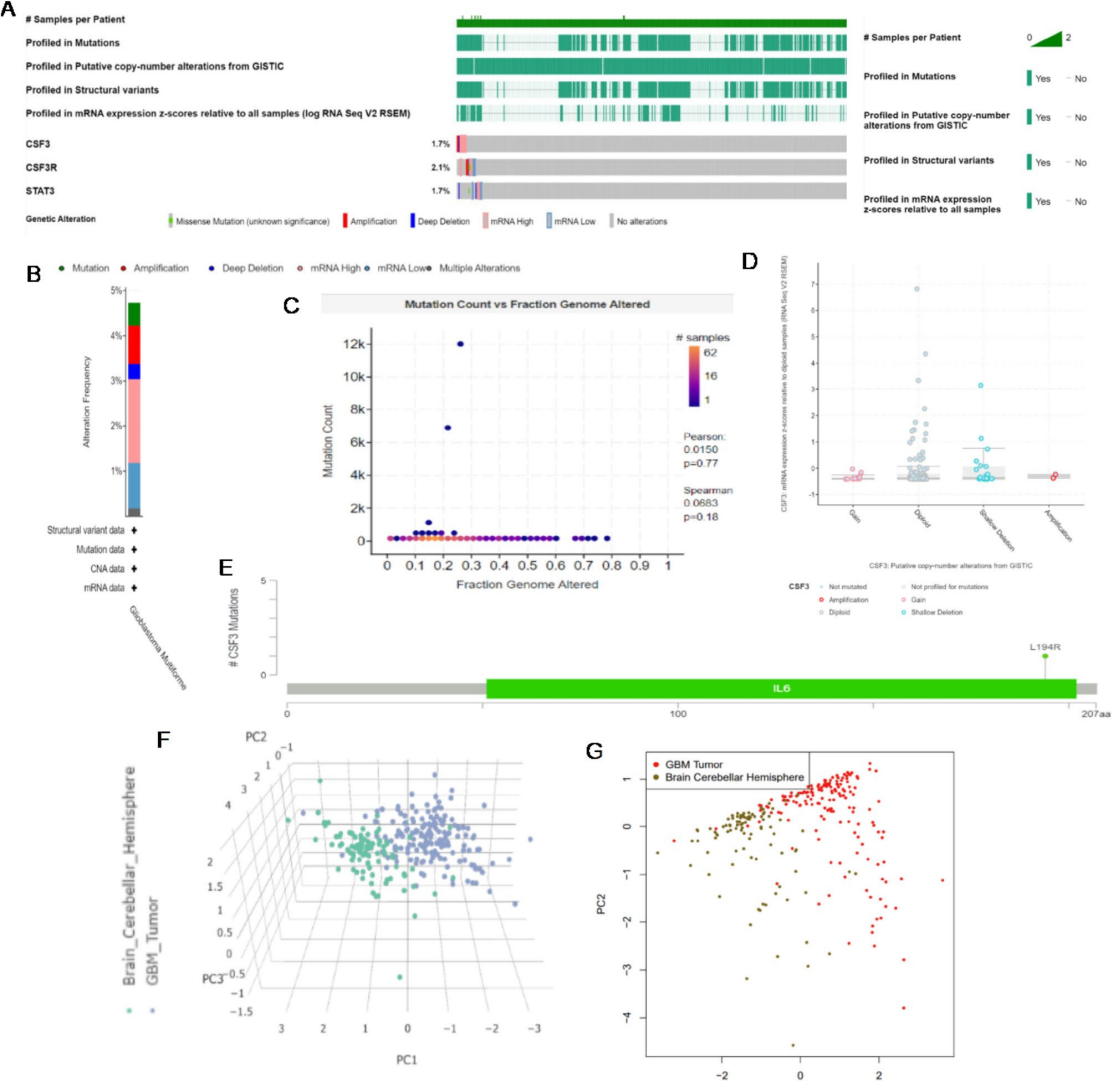
Genome landscape alterations in GBM patients (**A**) illustrates genetic alteration of GCSF 1.7%, GSCFR 2.1% and STAT3 1.7% (**B**) Alteration frequency in glioblastoma multiform depicts the percentage of mRNA low expression, high expression, deep deletion, amplification and mutation. (**C**) The comparison between the number of mutation counts and a fraction of the copy number alterations in the genome shows a positive Pearson and Spearman correlation with a significant p-value < 0.05. (**D**) GCSF gene amplification versus mRNA expression in GBM shows putative copy number alteration of diploid and shallow deletion according to GISTIC (Genomic Identification of Significant Targets in Cancer). (**E**) Distribution of GCSF mutations in GBM cancer across protein domains at hotspot (L194R) codons 100 and 207aa associated with elevated expression of IL6. (**F**) The scatterplot distribution of the first three principal components of PCA from the protein expression data differentiates between normal and malignant tissues used for PC analysis. The green dots represent the normal tissues, whereas the blue dots indicate tumours between ( +) and ( −) coordinates. (**G**) 2D plot also depicts the logarithmic log p-values for each protein on the x-axis and the y-axis showing GBM (red dots) projection between scale 0.5 to 1.5 of value.

We explored the potential mechanism of GCSF (CSF3), GCSFR (CSF3R) and STAT3 in the pathogenesis of GBM from the dataset of patient samples of the TCGA Pan-Cancer Atlas database with the respective genetic alterations of GCSF (CSF3) 1.7%, GSCFR (CSF3R) 2.1% and STAT3 1.7% respectively (Fig. 4A). According to the TCGA database, 4.73% of glioblastoma genes were found to be altered, with the following frequencies: mutation 0.51%, amplification 0.84%, deep deletion 0.34%, mRNA high 1.86%, mRNA low 1.0%, and multiple alteration 0.17% (Fig. 4B). The comparison between the number of mutations counts and a fraction of the copy number alterations in the genome shows the frequency of GCSF family genes mutation in GBM patient samples with significant positive Pearson and Spearman correlation of R = 0.77 and R = 0.18 respectively. In the case of each sample the fraction of the genome which altered with the number of mutations present (Fig. 4). We also used the cBioPortal and GEPIA2 database to examine frequency changes of GCSF mRNA expression (mRNA expression z-scores relative to diploid samples (RNASeqV2 RSEM)) including, shallow deletions, diploid, gain and amplifications upregulation of missense mutation. The findings suggest the median value ranges from − 0.39 to − 0.16 as low-level gain, diploid copy numbers between − 0.36 and 0.5 and included gain. The majority of them also showed few amplifications ranging from − 0.37 to − 0.24. However, shallow deletion showed a significant rise from − 0.36 to 0.73 (Fig. 4D). Another mutational analysis was also performed which depicts the position and frequency of all mutations within the framework of Pfam protein of respective homologous domains encoded by the highly expressed canonical isoform, as well as specific mutation positions. The length of the line linking the mutation annotations to the protein indicates the number of mutation-bearing samples. Germline frameshift mutations at hotspot (L194R) codons 100 and 207aa, associated with elevated IL6 expression, represent the majority of GCSF mutations as mentioned in Fig. 4E. The shared exclusivity study showed that distribution of cell cycle control was likely to occur in GBM through principal component analysis of GBM tumour cells in the brain.

To investigate the optimal gene combination to compare GBM with normal brain tissue, the combined expression levels of three validated genes (GCSF, GCSFR, and STAT3) were examined using PCA dimensionality reduction. According to Fig. 4F, the three-dimensional space represented by the three variance components (PC1, PC2, and PC3) attributed to the expression values of these three genes revealed a remarkable demarcation between 163 GBM and 207 healthy controls. The source data plot portraying the logarithmic log p-values for each protein reveals that the PCA-based component represents nearly all differential expressions (red Dots), keeping the GBM projection scale range from 0.5 to 1.5 as shown in Fig. 4G.

#### Prognostic value of differential abundances of infiltrative immune cells

Immune infiltration is often associated with developing a favorable tumor microenvironment for oncogenesis.

**Figure 5:**
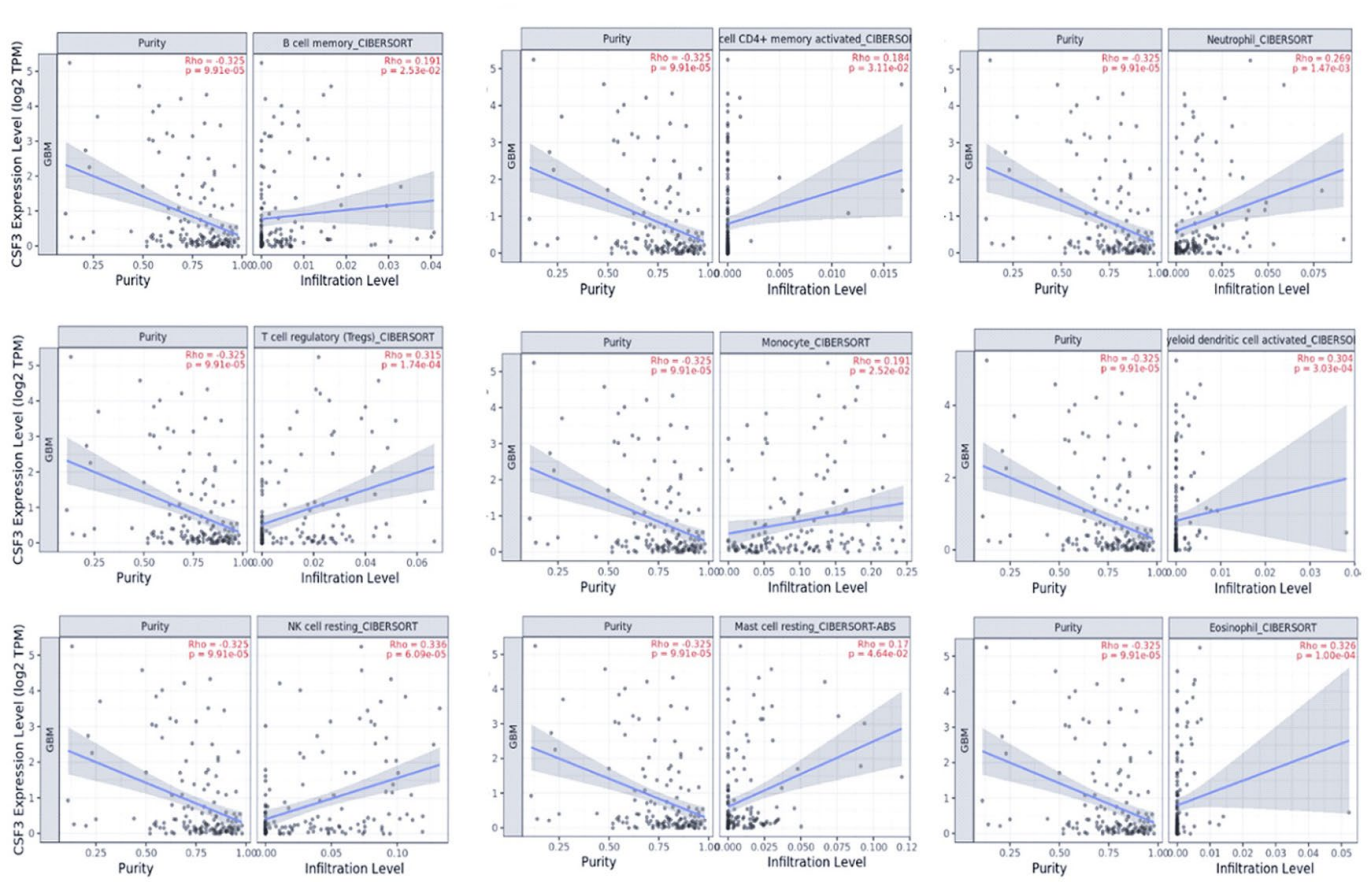
Correlation between GCSF expression with 09 immune infiltration levels using algorithms CIBERSOFT. The association between GCSF and immune infiltrating cells shows a positive differential infiltrative correlation with a significant p-value < 0.05.

In order to predict the prognostic value of expression of immune checkpoint signaling molecules and immune cell infiltration fractions in GBM. The TIMER database was used in the current investigation for systemic analysis of clinical impact of immune cells to examine the correlation between GCSF transcriptomic expression and its immune infiltration to suggest the effectiveness of newly developed immune checkpoint blockade medicines. The relative proportions of 09 different types of infiltrating immune cells, including T cells (CD4), Tregs, B cell memory, neutrophil, monocyte, myeloid dendritic cell, NK resting cell, mast cells, and eosinophils that were differentially expressed, were determined using the CIBERSORTx algorithm. These associations imply that several gene profiles linked to pro-tumor immune settings in GBM patients exhibit with differential GCSF expression. The significant expression of GCSF had a positive association with B cells (Rho = 0.191, p = 2.53e−02), CD4 + T cells (Rho = 0.184, p = 3.11e−02), Tregs (Rho = 0.315, p = 1.74e−04), neutrophils (Rho = 0.269, p = 1.47e−03), monocyte (Rho = 0.191, p = 2.52e−02), Myeloid dendritic cell (Rho = 0.304, p = 3.03e−04), NK cell resting (Rho = 0.336, p = 6.09e−05), mast cells resting (Rho = 0.17, p = 4.64e−02) and eosinophils (Rho = 0.326, p = 1.00e−04) as shown in Fig. 5.

#### GCSF co expression and enrichment analysis

**Figure 6:**
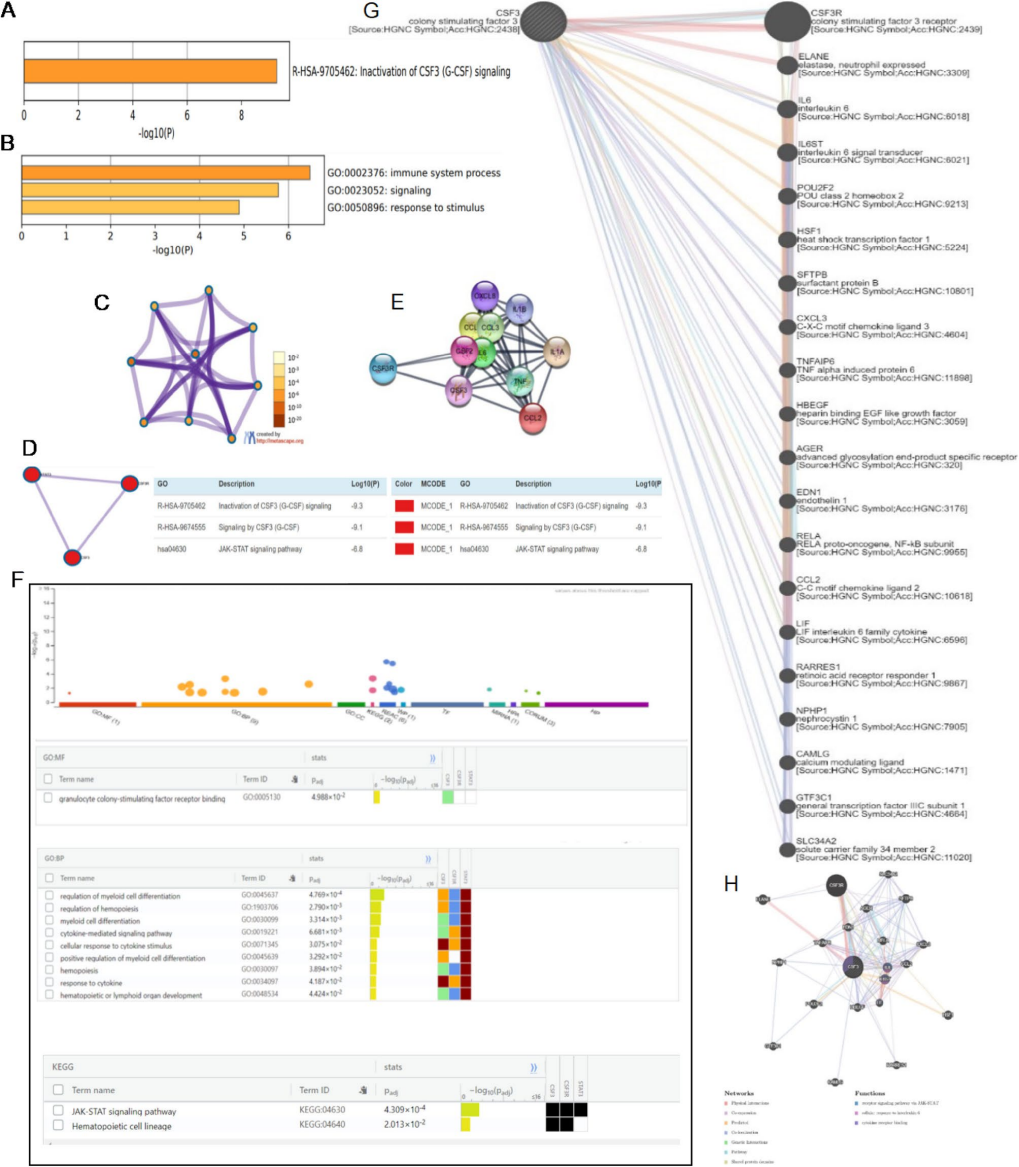
The enrichment analysis of GCSF (CSF3) in GBM patients (**A**) Ontology category clustering across the input gene CSF3 is illustrated using p-values from Metascape. Displaying Top 1 cluster (inactivation of CSF3 signaling) at log base-10 p-value. (**B**) Bar chart of the first three enriched terms for CSF3 also constructed through Metascape. (**C**) Enriched cluster analysis involving interacting genes based upon Color by cluster ID wherein nodes share the same cluster-ID are often adjacent and colored by p-value, similar genes have significant p-value. (**D**) Identification of enriched term net graph showed Module of molecular complex detection (MCODE) component. (**E**) protein–protein interaction (PPI) network of DEGs at significant p-values < 0.05. (**F**) Gene Ontology (GO) analysis of DEGs suggest essential molecular functions and comprise the crucial analysis of MF: molecular functions, BP: biological processes., CC: cellular components and KEGG: Kyoto Encyclopedia of Genes and Genomes pathway by showing (JAK-STAT signaling pathway and Hematopoietic cell linage) evaluated according to the levels of DEGs enriched in CSF3 at significant p-values < 0.05. (**G**) In GBM patients, the GeneMANIA database reported 20 genes significantly associated with CSF3 through gene–gene networks. (**H**) Differential mode of orientation of gene–gene network showing different functions of genes.

After Immune infiltration analysis, the molecular, cellular and biological functions of GCSF and the aforementioned co-expressed genes were also investigated using Metascape and g profiler. The enrichment background is comprised of all genes in the genome. During/ In investigation, the p-value < 0.01, and an enrichment factor > 1.5 were retrieved and clustered based on their association patterns. Furthermore, the most significant expressions integrating to the input set of genes were found using cumulative hypergeometric P-values by g: Profiler. In contrast, while performing hierarchical cluster analysis, q-values were determined using the Benjamini–Hochberg method. Then 0.3 Kappa scores were taken as the threshold, and sub-trees similarity > 0.30 were considered. The term having the highest statistical significance is selected to represent the cluster resulting in the inactivation of GCSF signaling at Log10 p = -9.30 (Fig. 6A). The most frequent terms are shown in the bar chart representing the p-value of immune system process, signaling and response to stimulus in response of GCSF, GCSFR, and STAT3 (Fig. 6B). In addition, a network map of the enriched terms with log p-values was also constructed as shown in Fig. 6C. In the protein interactions, densely connected network regions have been found using the Molecular Complex Detection (MCODE) algorithm. MCODE1 was associated with inactivation of CSF3 (GCSF) at Log10 p = -9.30, similarly the signaling of CSF3(GCSF) at Log10 p = -9.1, and JAK/STAT signaling pathway at Log10 p = -6.8 as shown in Fig. 6D. An enrichment study of protein–protein interactions of respective genes was also analyzed by using the STRING and BioGrid databases. The resulting network comprises the subset of proteins interacting physically with at least one other component (Fig. 6E). To further analyze the association and significant interactions between gene and disease, the genes were further analyzed by keeping Log p-value significant to estimate the molecular mechanism, signaling pathways, cellular and biological processes involved in glioblastoma. However, the Gene Ontology analysis of DEGs showed significant enriched terms (p-value < 0.05) of GCSF receptor binding as important molecular function. Similarly the significant biological process involved in regulation of myeloid cell differentiation and cytokine mediated signaling pathway while KEGG showed JAK/STAT signaling pathway at p-value < 0.05 significant in Fig. 6F. We evaluated the regulatory network and functionally related genes using the Gene MANIA database. We identified twenty (20) genes with the highest correlation, which were GCSFR, elastase neutrophil expressed (ELANE), Interleukin 6 (IL6), interleukin 6 signal transducer (IL6ST), POU Class 2 homeobox 2 (POU2F2), heat shock transcription factor 1 (HSF1), surfactant protein B (SFTBP), C-X-C Motif Chemokine Ligand 3 (CXCL3), TNF alpha induced protein 6 (TNFAIP6), Heparin Binding EGF Like Growth Factor (HBEGF), advanced glycosylation end-product specific receptor (AGER), endothelin 1 (EDN1), RELA proto-oncogene, NF-kB subunit (RELA), C–C Motif Chemokine Ligand 2 (CCL2), LIF Interleukin 6 family cytokine (LIF), retinoic acid receptor responder 1 (RARRES1), nephrocystin 1 (NPHP1),calcium modulating ligand (CAMLG), general transcription factor IIIC subunit1 (GTF3C1), solute carrier family 34 member 2 (SLC34A2) (Fig. 6G). The different modes of orientation from gene–gene network show the different functions of genes, including physical interaction at about 77.64%, and the group of genes which shows the physical interaction with each other e.g. GCSFR, GCSF, ELANE, TNFAIP6, AGER, RELA, IL6, IL6ST, LIF, CCL2 and the other gene functions include co-expression 8.01%, predicted 5.37%, colocalization of 3.63%, genetic alteration of 2.87%, pathway 1.88%, shared protein domain 0.60%, and pfam 0.21% (Fig. 6H).

#### KEGG pathway enrichment analysis and associated mechanisms

**Figure 7:**
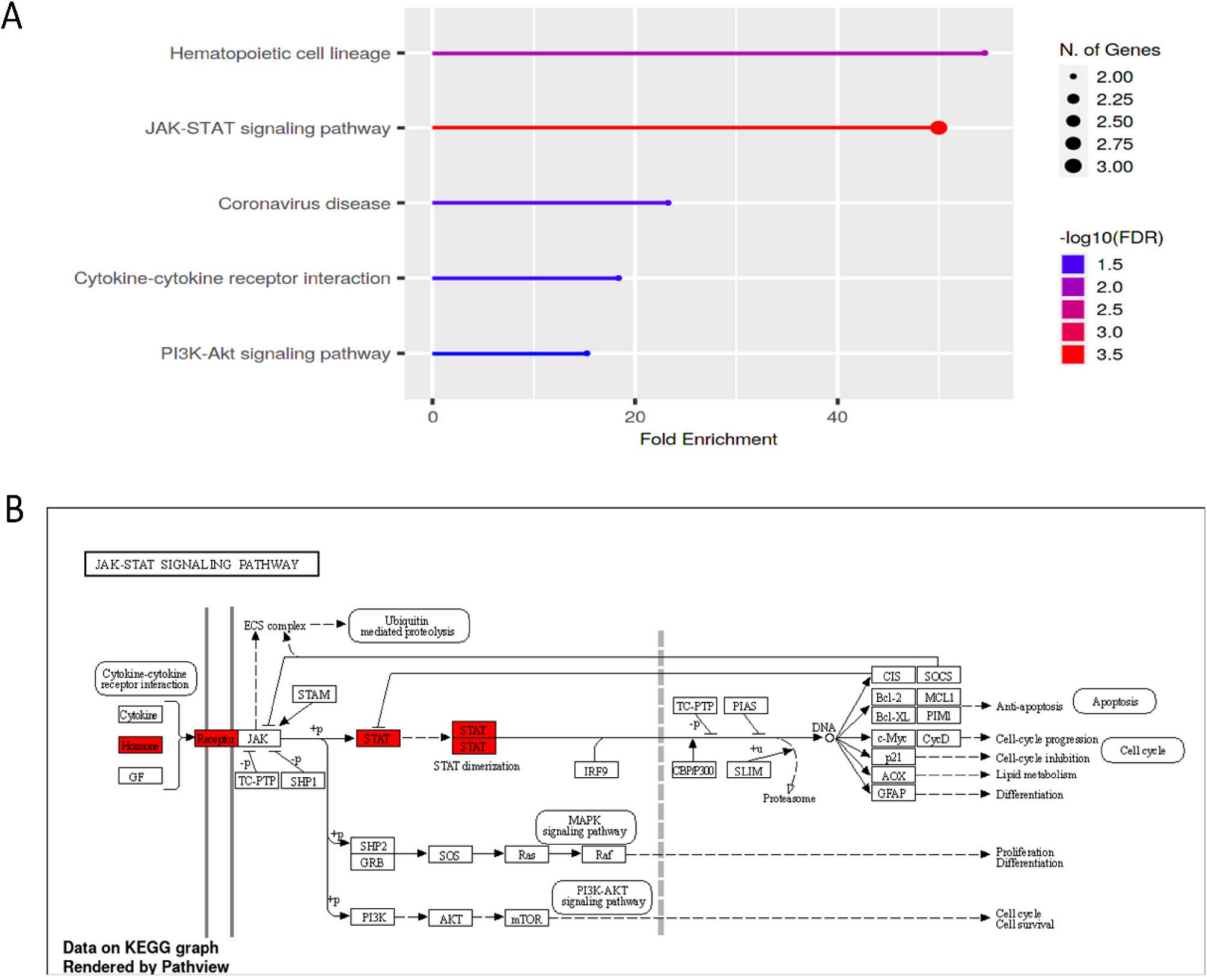
Pathway enrichment analysis (**A**) highlighting the percentage of DEGs in the biological pathway using the shiny GO tool. (**B**) KEGG and Wiki Pathways have been employed to map the pathways. Color codes are applied to explain the involvement of DEGs and their associated mechanism in the pathway model.

The GCSF, GCSFR, and STAT3 genes were also analyzed through fold chain enrichment analysis using shiny GO graphical enrichment tool, which identified five significant enriched pathways. The JAK/STAT signaling with maximum fold chain enrichment, while the association of hematopoietic cell lineage, Cytokine-cytokine receptor interaction, and PI3K-AKT signaling pathway with minimum value of fold chain enrichment as shown in Fig. 7A. The activation of STAT3 pathway is crucial to carcinogenesis and immune evasion. Various potential upstream and downstream regulatory mechanisms and development of the immunological milieu are transcriptionally upregulated by STAT3 activation. The equilibrium of cytokines stimulates the infiltration of immunosuppressive immune cell types and promotes STAT3 signaling within immune cell populations to induce immunosuppressive microenvironment. KEGG pathway indicates the genes involved in GBM progression showing significant fold chain enrichment of 3.5 –log10 (FDR) in JAK/STAT signaling pathway. We examined GCSF role in the associated pathways in GBM to highlight aberrant signal transduction cascades and potential drug targets especially in the critical JAK/STAT pathway (Fig. 7B).

#### Molecular docking of anticancer bacteriocins and validation by MTT assay

**Figure 8:**
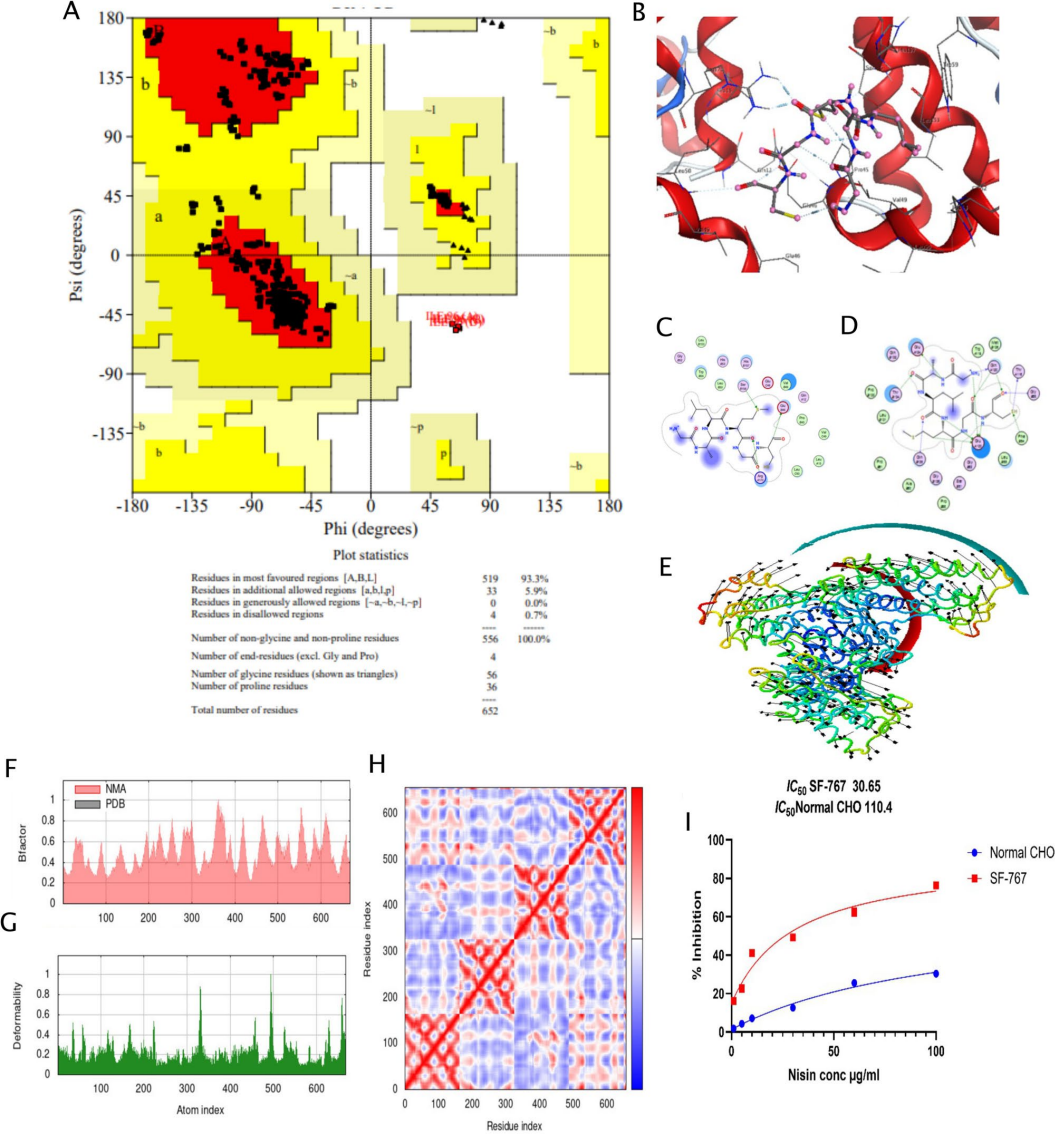
(**A**) Ramachandran plot statistics for modelled protein GCSF. (**B**) The best scoring docked complexes for target receptors 3D Docked pocket. It also demonstrates our ligand's overlaid 3D interaction with the active region of the receptor protein GCSF. The ligand molecule is shown in marine blue, the amino acid residues are shown in grey blue, and the protein structure is shown in red. (**C**) 2D interactions of 1WCO with 5GW9 reported 3 hydrophobic interactions with Ser B156, Glu46 and Arg 170 residues of cluster 0. (**D**) 2D interactions of 1WCO with 5GW9 reported revealed the 8 hydrophobic interactions with the Glu A124, Gln A120, Thr A116, Gly B88, Phe B84, Glu A123, Gln B135 and Thr B134 residues of cluster 1. (**E**) Molecular dynamics simulations of the complex generated between our drug and receptor, which represents the docked molecule orientation (**F–G**) represents outputs of NMA study deformability and B-factor plot graph. (**H**) represents residue index co-variance heat Map for the Dock modelled GCSF-Nisin. (**I**) The effects of Nisin on % inhibition as determined by MTT assay. Blue curve represents normal CHO and Red curve show SF-767 Cancer Cell lines which were incubated for 48 h with different concentrations of Nisin.

After expression Analysis in different integrated ways, the molecular docking was performed. For this, the crystal structure of C163, a backbone circularized GCSF of the model, was modified for further investigation. Various structure validation programmes evaluate to generate the model proteins, including stereochemical quality and geometrical conformations assessments. The Ramachandran plot computations were conducted using the PROCHECK software and revealed that 93.3% of residues were located in the most desirable zone, 5.9% in the permissible region, and 0.7% in the prescribed region of model protein as shown in Fig. 8A. In addition, the Rama favored region for the crystal structure of C163 was shown to be 93.3% for 5GW9. MOE software was used to dock anticancer bacteriocins peptide against GCSF in glioblastoma against a specific binding pocket. The energy function score (S-Score), hydrogen bonding, and the interaction with binding pocket were used to classify all complexes (Fig. 8B). Among 33 docked clusters, 7 were selected for interaction analysis based on highest levels of hydrogen bonding, van der Waals interaction, and other hydrophobic interactions with binding pocket residues. Cluster 0 had the lowest energy-weighted score of − 554.7 kcal/mol with a total 413 members, Moreover, the visualization of the docking results reported 3 hydrophobic interactions with Ser B156, Glu 46 and Arg 170 residues of cluster 0. Furthermore, cluster1 had a score of -538.1 kcal/mol and their visualization revealed the 8 hydrophobic interactions with the Glu A124, Gln A120, Thr A116, Gly B88, Phe B84, Glu A123, Gln B135 and Thr B134 residues (Fig. 8C−D). The stability and the quality of the docked model was examined by dynamic simulation through deformability and B-factor as shown in (Fig. 8E).

The results indicated an insignificant hinge, and the B-factor provides an average RMS value, as the experimental B-factor was used to determine the protein mobility changes associated with protein–ligand complex interactions and calculated from NMA method (Fig. 8F). The degree to which a given molecule can deform at each of its residues is known as deformability. This is primarily seen as "highest peaks" that can be produced in high deformability spots. The docked NISIN complex exhibits a small amount of deformability since it has peaks with deformability indices that range from about 0.8 to 1.0. A low likelihood of deformation was suggested for the anticipated homology model (Fig. 8G). The correlation between the residues in the complex is shown by the covariance matrix, the stronger the correlation, the better the complex. White color illustrates no correlation, while blue coloration shows anticorrelations. Red coloration denotes a strong correlation between residues. The Nisin docked complex predicts a strong correlation with only a few anticorrelations (Fig. 8H). The dose-dependent cytotoxicity of Nisin against SF-767 cells and normal cell line CHO was evaluated using MTT assay and determined by the statistical analysis.

In this study the proliferation of SF-767 cell was significantly inhibited showed 78.15% inhibition at 100 µg/mL while at 1 µg/mL was 16.70%. At 48 h. the IC50 value of Nisin for SF-767 was 30.65 µg/mL found. However, the results demonstrate that Nisin has lower cytotoxicity in a normal cell line, CHO, with an IC50 value of 110.4 g/mL as compared to glioblastoma cell line, SF-767, as shown in (Fig. 8I).

## Discussion

Glioblastoma multiforme (GBM) is a highly heterogeneous tumor at the genomic level revealing the intricate and complex molecular identity^51^. The priority of molecular characterization for glioma subtyping is emphasized in the recent CNS 5 WHO classification of brain tumours. However the presence of IDH wild-type (WT) gene classify a glioma as a glioblastoma (CNS WHO grade 4)^52^. The main objective of the current study was to investigate the role of GCSF (CSF3) in GBM, particularly the underlying regulatory molecular mechanism through comparative analysis. In this regard, we analyzed GCSF, GCSFR and STAT3 gene expressions between tumour-associated normal tissue (TANT) and IDH-wild type, (CNS WHO grade 4) glioma (GBM) resulting in statistically significant differences in their expressions. The co-expression of GCSF and GCSFR indicates that it may heighten autocrine and paracrine signaling in GBM^11^. Granulocyte macrophage colony stimulating factor (GMCSF), commonly known as CSF2, has been the subject of numerous expression investigations in the past. G-CSF and GM-CSF may correspond for a variety of roles but also apposing to perform some functions^53,53,53^. The originality of the present study, however, is in the first-time reporting of a comparative analysis of the differential expression of GCSF (CSF3) in Pakistan. It was shown through bioinformatics analysis of publicly accessible TCGA data for GCSF (CSF3) that GCSF expression was not consistent and significant in GBM, however earlier research had found increased GCSF and G-CSFR expression in glioma samples from the Chinese population^55^. Our findings thus confirmed the widespread expression of GCSF in Pakistan glioblastoma patients in the similar pattern, however the difference did not surpass log2FC (fold change). According to our findings, ethnicity and genetics may possibly have a role in the varied expression of GCSF in glioblastoma patients. However, meta-analysis of earlier studies revealed a link between polymorphism and the pathogenicity of gliomas. For instance, the Arg399Gln polymorphism was linked to a higher risk of GBM in Caucasians and glioma in Asians. However, Arg194Trp/Arg280His, polymorphisms most likely have no impact on gliomas in various ethnic groups. Patients with inherited tumor syndromes, such as Turcot syndrome and Li-Fraumeni syndrome, have a higher prevalence of GBM. Otherwise, GBM happens infrequently and without a genetic predilection that is known^56^. The glioblastoma patients biopsy samples RT-PCR based study revealed that GCSF modulated the malignant biologic properties of glioma cells as a tumor-promoting factor. The results of increased expression of GCSF, GCSFR and STAT3 genes were validated by quantifying their respective proteins through ELISA. Constitutive STAT3 activation suppresses host anti-tumor immune responses, facilitating unregulated tumorigenesis, angiogenesis and induction of immune evasion mechanisms. In the current study, STAT3 activation seems to be facilitated by increased expression of GCSF. And showed a positive correlation among GCSF and STAT3 of GBM biopsies while GCSF and STAT3 showed weak correlation with TANT. STAT3 expedites GBM immune evasion via a decrease in activated circulating lymphocytes and a decrease in Tregs^57^. The system-level framework approach endorsed the meta-analysis of RNA-seq datasets identifying differentially expressed genes^58^. Thus, our results demonstrated that though histopathologically CNS WHO grade 4 IDH wildtype glioblastoma malignancy is a single entity, their molecular mechanisms differ distinctly based on expressions of specific genes. The complex interplay between glioblastoma's imaging traits, histological properties, and gene expression patterns reveals the biology and heterogeneity of the tumor. Magnetic resonance imaging and histological results were used in this investigation to characterize GBM tumors. (Fig. 2A,B). Necrosis-affected areas, contrast enhancement, edema, and uneven boundaries are significant characteristics. When compared to normal tissues, the histopathology revealed atypia and darker staining, as well as a high proliferation index, localised necrosis, hyperchromatic tumor cells, and dense cellularity. (Fig. 2C,D). Histopathological observations of necrotic and enhanced cellularity generally match imaging characteristics of glioblastoma, such as areas of necrosis and contrast enhancement. These associations confirm that imaging accurately depicts the underlying cellular and structural alterations in the tumor^59^. To evaluate the potential role of GCSF in glioblastoma, we quantified the expression of GCSF and its receptor (GCSFR) and STAT3. Gene expression analyses through RTPCR and ELISA have revealed differential expression within glioblastoma based on molecular signatures. Different molecular subtypes, such as classical, mesenchymal, proneural, and neural, have been found by the Cancer Genome Atlas project. Each is associated with distinct genetic abnormalities and clinical consequences. In glioblastoma biopsy samples, GCSF mRNA and protein expression were significantly elevated, and particular imaging features such contrast enhancement and necrotic areas frequently correlated with hypoxia areas in the tumour microenvironment. The gene expression of targeted genes has been examined among glioblastoma specimen sections within tumors and tumor-associated normal tissue (TANT). GCSF, GCSFR, and STAT3 exhibited increased expression in tumor tissue biopsy samples. Specific imaging characteristics, like contrast enhancement, can be associated with increased angiogenesis, which is driven by upregulated genes involved in vascularization pathways^60^. Additionally, areas of necrosis observed on imaging often correspond to hypoxic regions in the tumor microenvironment, which can be linked to altered gene expression related to hypoxia-responsive pathways^61^. Molecular subtypes and certain genetic alterations, identified through gene expression analysis correlate with histopathological characteristics with increased cell invasion, reflecting the infiltrative tumor edges^62^.

These results revealed that high GCSF and GCSFR expressions were independent predictors of decreased OS for GBM. Overall, our research provides real-world data to confirm that both genes are positively associated with immunosuppressive and tumour-promoting phenotypes and negatively associated with patient survival. In the current study Immune microenvironment-related bioinformatic algorithms have been applied for the better prognosis of GBM. The effectiveness of this algorithms was also reported in the previous studies related to gliomas, breast cancer, and cervical cancer. To better understand the proportions of immune cells in the TME, The CIBERSORT (Cell type Identification By Estimating Relative Subsets Of RNA Transcripts) deconvolution software has been used^63^. Infiltrating immune cells in GBM have been evaluated for their prognostic significance. Nine different types of heavily infiltrating immune cells were found to be predictive of longer overall life, with the majority of these findings having been supported by earlier research. Numerous studies highlight GCSF-mediated exacerbation of inflammation in the tumor microenvironment. In agreement with previous findings, various studies validate the aggravated expression of GCSF in multiple cancer types^64^.

The oncogenic mechanisms associated with GBM progression include changes to the genetic sequence and karyotype, with some events resulting in chromosomal aberrations^65^. In the current finding, the GCSF gene encodes deep deletions (0.34%), mutations (0.51%), amplifications (0.84%), mRNA high 1.86%, mRNA low 1.01%, and multiple alterations 0.17%. Correlations among mutations and the fractions of copy number are directly proportional to the significantly upregulated mRNA expression^66^.

The heterogeneity that influences the tumour immune microenvironment remains elusive^67^. GBM samples contained nine immune cells associated with survival. Previous research has shown that immune cells, particularly tumor-associated macrophages, interact with tumor cells through direct contact or various signaling pathways^68,68^. These findings suggest potential GCSF (CSF3) signaling associations with how TME is shaped and maintained, potentially affecting responses to immunotherapies. Many studies have reported that the co-expression of GCSF and IL-6 involved co-augmenting effects on neutrophils, including an elevation in STAT3 expression and diminution in JAK/STAT pathway activation^10,69^. These differential immune-related genes are enriched in the JAK/STAT signaling pathway based on the findings of our study.

In the case of protein–protein interactions, we identified that these potential drug targets are intricately interconnected with other associated target proteins. The normal expression patterns of respective DEGs affect the expression of these biomolecules, while anomalies result in dysregulation of the pathways that control their expression. Previous research investigations have shown that GCSF increased STAT3 phosphorylation and JAK2 overexpression via binding to G-CSFR, which may foster GBM cells migration and proliferation^70^. Filgrastim (rhG-CSF) is administered parenterally to ameliorate chemotherapy induced neutropenia. This study shows that GCSF can upregulate GCSFR and STAT3 in glioblastoma patients. Therefore, further studies are needed to investigate whether administering rhGCSF following chemotherapy lowers drug effects by upregulating GCSFR-positive tumor growth. However, we found that it is highly expressed in high-grade glioblastoma suggesting a strong association between GCSF and GCSFR, causing STAT3 activation by increased phosphorylation.

The characteristics of cationic peptides, including Nisin enable them to induce apoptosis in tumor cells^71^. Therefore, they are increasingly regarded as potential agents for improving anticancer therapy. A ligand with minimal binding energy is preferred because a low binding score is directly associated with higher binding affinity. The docking study of Nisin with GCSF (5GW9) protein reveals 33 docked clusters, 7 were used as the best for hydrophobic interactions. Cluster 0 had the lowest energy-weighted score of − 554.7 kcal/mol and gave the least complex energy. This study identified that the hydrogen bond interaction of the anticancer peptide (Nisin) drug with the amino acid residues was potent in both clusters, and the overall docked complex attained a stable conformation on these interactions. In the current study, Nisin elicited significant apoptosis in the SF-767 cell line compared to the normal CHO cell line. The significance of SF-767 has been also reported in the previous study^72^. Tumor cells are negatively charged because their cell membranes include anionic substances like phosphatidylserine. Normal cells have a neutral charge due to their zwitterionic lipid membranes. Electrostatic interactions between cationic Nisin and cancer cell membranes may contribute to its selectivity. Our findings reveal that the concentration of Nisin has a direct effect on cell viability. It has been reported that Nisin induced programmed cell death, arrest of the cell cycle and inhibited HNSCC cell growth in contrast to the normal cell line^73^. Another study found that Nisin had a cytotoxic effect on colorectal cancer cells via intrinsic mechanisms responsible for apoptosis. This study systematically investigated the expression levels of GCSF, GCSFR, and STAT3 using the TCGA database and glioblastoma tissue biopsy samples. However, the most significant limitation of the current study was its retrospective nature. Further studies can elucidate the regulatory mechanisms of GCSF and GCSFR in GBM progression. Moreover, in vivo experiments are needed to elucidate the molecular mechanisms of interacting GCSF, GCSFR, STAT3 and the therapeutic potential of Nisin against glioblastoma^74^.

## Conclusion

This study identified a functional expression profiling of GCSF, GCSFR, and STAT3 for GBM prognosis in Pakistan Elevated GCSF expression levels correlate with increased GCSFR expression levels and GBM associated JAK/STAT signaling pathway. We examined 21 biopsy samples gene expression of glioblastoma patients, which showed elevated expression of G-CSF, G-CSFR, and STAT3 at the transcriptome and proteomic levels compared to tumor-associated normal tissue (TANT), which might be considered diagnostic and prognostic biomarkers. Furthermore, comparative transcriptome profiling of GCSF, CGSFR, and STAT3 genes in GBM (163 cases) and normal tissue samples (207 cases) from integrated genome atlas databases also demonstrated the genetics changes, functional annotation and immune cell infiltration, resulting differential expression of genes and reduced overall survival. This study also revealed distinct immune infiltration patterns through bioinformatics analysis and indicated that immunomodulators are essential determinants of GBM prognoses. The computational docking of potential therapeutic candidate Nisin against GCSF was also performed, and the results were verified in vitro through MTT assay using a glioblastoma cell line SF-767. These findings imply that G-CSF contributes to glioblastoma progression and there blocking by using the anticancer bacteriocin peptide Nisin may dampen tumor progression and will have better therapeutic response as angiogenesis inhibitors.

## Data Availability

The datasets generated during and/or analyzed during the current study are available from the corresponding author on reasonable request.
